# Cdc14 Early Anaphase Release, FEAR, Is Limited to the Nucleus and Dispensable for Efficient Mitotic Exit

**DOI:** 10.1371/journal.pone.0128604

**Published:** 2015-06-19

**Authors:** Christopher M. Yellman, G. Shirleen Roeder

**Affiliations:** 1 Howard Hughes Medical Institute, Chevy Chase, Maryland, 20815, United States of America; 2 Department of Molecular, Cellular and Developmental Biology, Yale University, New Haven, Connecticut, 06520, United States of America; 3 Department of Genetics, Yale University, New Haven, Connecticut, 06520, United States of America; Stony Brook University, UNITED STATES

## Abstract

Cdc14 phosphatase is a key regulator of exit from mitosis, acting primarily through antagonism of cyclin-dependent kinase, and is also thought to be important for meiosis. Cdc14 is released from its sequestration site in the nucleolus in two stages, first by the non-essential Cdc Fourteen Early Anaphase Release (FEAR) pathway and later by the essential Mitotic Exit Network (MEN), which drives efficient export of Cdc14 to the cytoplasm. We find that Cdc14 is confined to the nucleus during early mitotic anaphase release, and during its meiosis I release. Proteins whose degradation is directed by Cdc14 as a requirement for mitotic exit (e.g. the B-type cyclin, Clb2), remain stable during mitotic FEAR, a result consistent with Cdc14 being restricted to the nucleus and not participating directly in mitotic exit. Cdc14 released by the FEAR pathway has been proposed to have a wide variety of activities, all of which are thought to promote passage through anaphase. Proposed functions of FEAR include stabilization of anaphase spindles, resolution of the rDNA to allow its segregation, and priming of the MEN so that mitotic exit can occur promptly and efficiently. We tested the model for FEAR functions using the FEAR-deficient mutation *net1-6cdk*. Our cytological observations indicate that, contrary to the current model, FEAR is fully dispensable for timely progression through a series of anaphase landmarks and mitotic exit, although it is required for timely rDNA segregation. The *net1-6cdk* mutation suppresses temperature-sensitive mutations in MEN genes, suggesting that rather than activating mitotic exit, FEAR either inhibits the MEN or has no direct effect upon it. One interpretation of this result is that FEAR delays MEN activation to ensure that rDNA segregation occurs before mitotic exit. Our findings clarify the distinction between FEAR and MEN-dependent Cdc14 activities and will help guide emerging quantitative models of this cell cycle transition.

## Introduction

Release of Cdc14 phosphatase from the nucleolus is a critical event during mitotic exit in the budding yeast *Saccharomyces cerevisiae* [1]. Cdc14 counteracts mitotic cyclin-dependent kinase (Cdk) activity by dephosphorylating and thereby activating three substrates: Sic1, a direct Cdk inhibitor, Swi5, a transcription factor for Sic1, and Cdh1, a substrate-specificity factor for the ubiquitin ligase APC^Cdh1^ [2, 3]. APC^Cdh1^ subsequently targets key mitotic proteins for degradation, including the major mitotic cyclin Clb2 and the mitotic kinase Cdc5, permitting the transition to G_1_ phase.

Cdc14 activity is strongly influenced by localization. From G_1_ through metaphase, Cdc14 is bound by the protein Net1 in a nucleolar complex called RENT (Regulator of Nucleolar silencing and Telophase exit). Release of Cdc14 from the RENT complex and from the nucleolus is generally equated with activation [4–6], but at least one report suggests that Cdc14 can be active while bound to the RENT complex [7].

Two distinct pathways are competent to release Cdc14 from Net1. During early anaphase, Cdc14 is transiently released from the nucleolus by the Fourteen Early Anaphase Release (FEAR) pathway [8, 9]. In late anaphase, Cdc14 is released in a sustained manner by the Mitotic Exit Network (MEN), promoting full exit from mitosis [10]. In addition to releasing Cdc14 from the nucleolus, the MEN drives export of Cdc14 to the cytoplasm, a critical requirement for mitotic exit [11]. The MEN kinase Dbf2-Mob1 stimulates nuclear export of Cdc14 by direct phosphorylation of Cdc14 at residues that inhibit its nuclear localization signal. As cells enter G_1_ phase following exit from mitosis, Cdc14 completes its localization cycle by returning to the nucleolus rapidly and efficiently [12].

FEAR-dependent release of Cdc14 requires phosphorylation of Net1 by mitotic Cdk, which disrupts the interaction between Net1 and Cdc14 [13]. Before early anaphase, FEAR is also subject to inhibition by protein phosphatase 2A (PP2A), which when coupled with its B-type subunit Cdc55, counteracts Cdk by dephosphorylating Net1 [14, 15]. Therefore, the activation and timing of FEAR are largely determined by control of the antagonistic actions of Cdk and PP2A^Cdc55^ on Net1.

A complex and poorly understood pathway has been proposed to trigger FEAR by linking the release of Esp1 (separase) at metaphase to downregulation of PP2A^Cdc55^ [14]. Esp1 has a well-understood function in the transition from metaphase to anaphase, specifically proteolysis of the sister chromatid cohesin protein Mcd1 [16], which initiates anaphase sister chromatid separation and subsequent segregation. It has been proposed that Esp1 has an additional, non-proteolytic activity, in concert with the kinetochore protein Slk19 and the proteins Zds1 and Zds2, which leads to down-regulation of PP2A^Cdc55^ [14, 17, 18]. It is important to note that the evidence for the role of this pathway in anaphase spindle regulation has been generated by using overexpression of Esp1, Zds1 or Zds2 in metaphase-arrested cells to trigger Cdc14 release [17, 19, 20]. More recent findings challenge this model by demonstrating that Esp1 contributes to mitotic exit primarily through the destruction of sister chromatid cohesion in a manner that requires mitotic spindle activity [21]. It appears, therefore, that Esp1 might activate mitotic exit by permitting spindle growth and spindle pole translocation into the daughter bud, a potential mechanism for activation of the MEN [22]. Additional recent work indicates that Zds1 and Zds2 downregulate PP2A^Cdc55^ by stimulating the nuclear export of Cdc55, allowing Net1 phosphorylation to accumulate [23, 24].

It has been suggested that Cdc14 release by the FEAR pathway might be limited to the nucleus [21], or alternatively that there could be some export to the cytoplasm [8]. Current evidence concerning Cdc14 localization during early anaphase is divergent. Most images indicate a largely nuclear localization [9, 15]. However, some studies of Esp1, Zds1 or Zds2 overexpression show Cdc14 being released to both the nucleus and cytoplasm [17, 18]. Export from the nucleus would allow Cdc14 access to mitotic exit regulators such as Swi5, Cdh1 and Cdc15 in the cytoplasm, at the bud neck or on the cytoplasmic face of the spindle pole [22, 25–28]. Therefore, it would be useful to know whether the FEAR pathway can stimulate nuclear export of Cdc14.

Spo12, a protein of unknown biochemical activity, is thought to contribute to FEAR by promoting dissociation of Cdc14 from the RENT complex member Fob1 [7, 9]. However, increased dosage of Spo12 has also been shown to suppress MEN defects [29–31].

It would be useful to know whether the activation of FEAR alone could contribute directly to the degradation of any key mitotic regulators. Conversely, if FEAR activity alone does not initiate degradation of these key proteins, then elevated levels of Spo12 might work through an additional FEAR-independent activity that promotes mitotic exit. This would indicate an additional, FEAR-independent function for Spo12.

Current assays of Cdc14 localization rely largely upon standard indirect immunofluorescence microscopy [7] or live-cell imaging of Cdc14-fluorescent protein fusions Lu, 2010 #72}. It is difficult to distinguish Cdc14 release to the nucleus alone vs. release to the nucleus and cytoplasm by these methods. We applied image deconvolution to indirect immunofluorescence microscopy, the highest resolution method currently available, to make this distinction clear. We tested the prediction that Cdc14 released by the FEAR pathway is limited to the nucleus. We also stabilized FEAR-released Cdc14 using a novel mitotic arrest protocol to accumulate cells that had fully activated FEAR, but not the MEN. This arrest method allowed us to clearly distinguish the two pathways, and ask whether sustained FEAR could stimulate mitotic exit activities such as nuclear export of Cdc14 or the degradation of key mitotic proteins. We find that Cdc14 released by the FEAR pathway is limited to the nucleus in both mitosis and meiosis I, and that even prolonged FEAR activation does not lead to the protein degradation characteristic of mitotic exit.

FEAR is believed to contribute to mitotic exit by priming the MEN to make mitotic exit more efficient, but the mechanism of this putative effect is poorly understood [8, 9]. One caveat to using FEAR mutations such as *slk19Δ*, *spo12Δ* and conditional mutations in *ESP1* to examine the role of FEAR in mitotic exit is that the protein products of these genes may have unknown functions in addition to their roles in FEAR, and these functions might affect mitotic exit. Accordingly, we investigated the functional importance of FEAR in mitotic exit using the *net1-6cdk* mutation, which eliminates the six Cdk phosphorylation sites in Net1 that are required for FEAR [13], blocking FEAR at the last known step in its activation. The *net1-6cdk* mutation has been shown to prevent Cdc14 early anaphase release and to delay rDNA segregation as severely as the canonical FEAR mutation *spo12Δ*, without conferring any obvious spindle defect [13].

The FEAR pathway model and its connection to mitotic exit have been established exclusively through experiments performed using the budding yeast *Saccharomyces cerevisiae*. In contrast to those findings, the *Schizosaccharomyces pombe* (fission yeast) orthologs of the FEAR pathway members Cdc5, Esp1, Slk19 and Spo12 do not promote the release of the *S*. *pombe* Cdc14 ortholog Clp1/Flp1 from the nucleolus [32]. Furthermore, while *S*. *cerevisiae* FEAR is thought to be required for efficient resolution of rDNA (nucleolar DNA) cohesion during anaphase [33], Clp1/Flp1 is not required for nucleolar segregation [32]. The lack of either a definitive requirement for FEAR or of functional conservation of the pathway members prompted us to re-examine the assumptions of the model that has placed FEAR at the center of so many critical anaphase events.

In an unexpected reversal of the current FEAR model, we find that the *net1-6cdk* mutation, although it severely compromises the early anaphase release of Cdc14, actually suppresses loss of function mutations in the MEN pathway. We also find FEAR to be dispensable for timely progression through anaphase and timely MEN-driven Cdc14 localization cycling. Our findings indicate that FEAR does not contribute to mitotic exit, and suggest that it may instead subtly restrain MEN activity while rDNA segregation is completed.

## Materials and Methods

### Yeast strains

S1 Table lists the names of the *S*. *cerevisiae* strains used in this study and their full genotypes. All strains were of the W303a background except four A364a strains (noted in the table with their strain names). Gene deletion alleles were transferred by PCR from a yeast gene deletion strain (Open Biosystems) and confirmed by PCR following integration, or have been previously published. Epitope fusions were made by standard methods [34, 35] and confirmed by genomic DNA sequencing. The *net1-6cdk* mutation was transferred from the Deshaies lab strain RJD 2862 by a *delitto perfetto* strategy [36] and confirmed by genomic DNA sequencing. Growth and genetic manipulation of yeast were carried out using standard media and methods [37]. The specific strains used in each figure and table are listed below.

#### Yeast strains used by figure and table

S2 Table lists, by relevant genotype and strain name, the strains used in each figure, table and accompanying genetic experiments.

### Mitotic growth, synchronization and nocodazole-induced arrest

To synchronize cells, mid-log phase cultures at a density of ~1 X 10^7^ cells/ml, growing in YPAD liquid at 30°C, were arrested by addition of 0.1 μM α-factor. Cells were allowed to accumulate in G_1_ phase for 2 hours and 20 minutes, washed twice with 50 ml of YPAD in a vacuum filter and released into fresh liquid growth medium. All synchronous cultures were incubated at 25°C and α-factor was re-added to the cultures after bud emergence to limit cells to a single mitotic cycle. For nocodazole-induced arrest, cells were released into YPAD containing 12.5 μg/ml nocodazole (10 mg/ml stock in DMSO), and for normal growth, cells were released into YPAD. For all experiments requiring synchronous arrest and release, bud morphology was monitored to confirm synchronization, timely release, and maintenance of mitotic arrest (S2 Fig).

### Induction of meiosis

The liquid sporulation medium used has been described [38]. To induce entry into meiosis, cells were grown at 30°C to early stationary phase (1–2 X 10^8^ cells/ml) in YPAD medium, washed once with water, diluted to an OD_600_ of 1.0 in fresh sporulation medium and incubated at 30°C on a platform shaker. The time of transfer to sporulation medium was considered the zero time point, and cells used for imaging meiosis were sampled 10–12 hours later.

### Determination of nuclear division stages

For stage-specific analysis, cells undergoing nuclear division were divided into the following categories: metaphase (short, thick spindle with round DNA mass), early anaphase (spindle and DNA mass slightly elongated), mid anaphase (intermediate length spindle and elongated DNA mass), late anaphase (fully elongated spindle with a weakened mid-zone and the majority of DNA in two separate masses), telophase (disassembled spindle with two fully distinct DNA masses). Note that the intranuclear portion of the spindle was used to determine cell cycle stage. Meiosis I and II were distinguished by the presence of one or two spindles, respectively.

### Whole-cell immunofluorescence

Cells were fixed in growth medium with 3.7% formaldehyde at room temperature for 30 minutes. Mitotic cells were digested in SCE (1 M sorbitol/0.1 M sodium citrate/10 mM EDTA) with 20 μg/ml zymolyase (100T, ICN Biomedicals). Meiotic cells were digested with a solution containing 50 μg/ml zymolyase and 1:50 glusulase (Perkin Elmer). Cells were mounted on poly-L lysine coated slides and proteins detected by indirect immunofluorescence.

Immunostaining of whole cells was as follows: Cdc14-Myc7 fusion protein was detected with mouse monoclonal anti-Myc 9E10 antibody (Covance) at 1:600 in PBS/1% BSA for 3 hours at room temperature, followed by anti-mouse CY3 (Jackson ImmunoResearch) secondary antibody at 1:600 for 1 hour at room temperature. Slide preparation was finished by adding mounting medium containing DAPI. Detection of tubulin was done with the rat monoclonal antibody YOL 1/34, followed by anti-rat FITC (Jackson ImmunoResearch).

### Protein localization imaging and image deconvolution

Immunofluorescence images were taken using an Olympus UPlanS APO 100X objective lens (numerical aperture 1.40) on an Olympus IX-70 microscope equipped with the DeltaVision RT (Applied Precision) imaging system. Z-section image series were collected at 0.2 μM intervals over a total of 3–4 μM through the center of the cells and deconvolved using softWoRx (Applied Precision).

### Quantification of Cdc14 localizations

Standard immunofluorescence microscopy, using a Nikon E800 microscope with a 100x objective (numerical aperture 1.40), was used to count cells showing the different Cdc14 localizations. For time course experiments, one hundred cells were assayed at each time point. Nucleolar heterochromatin is partially refractory to DAPI stain, leading to a weakened fluorescent signal relative to the rest of the nuclear DNA. Cdc14 was determined to be nucleolar when it was tightly localized to a part of the nucleus with weak DAPI signal and nuclear when its distribution broadened to include the strongly DAPI-stained area. All quantification of Cdc14-7Myc localization in meiosis was done using deconvolved images.

### Growth assay of serial dilution series

Cells were grown to saturation in liquid YPAD at 23°C and a 10-fold dilution series of the saturated cultures was prepared in water from 10^1^ to 10^6^, A multi-channel pipette was used to plate 5 μl of each dilution was plated onto YPAD agar medium, and the plates were incubated at the indicated temperatures for 2–3 days until colony growth

## Results

### Nuclear-limited Cdc14 release in normal cycling cells

**Fig 1:**
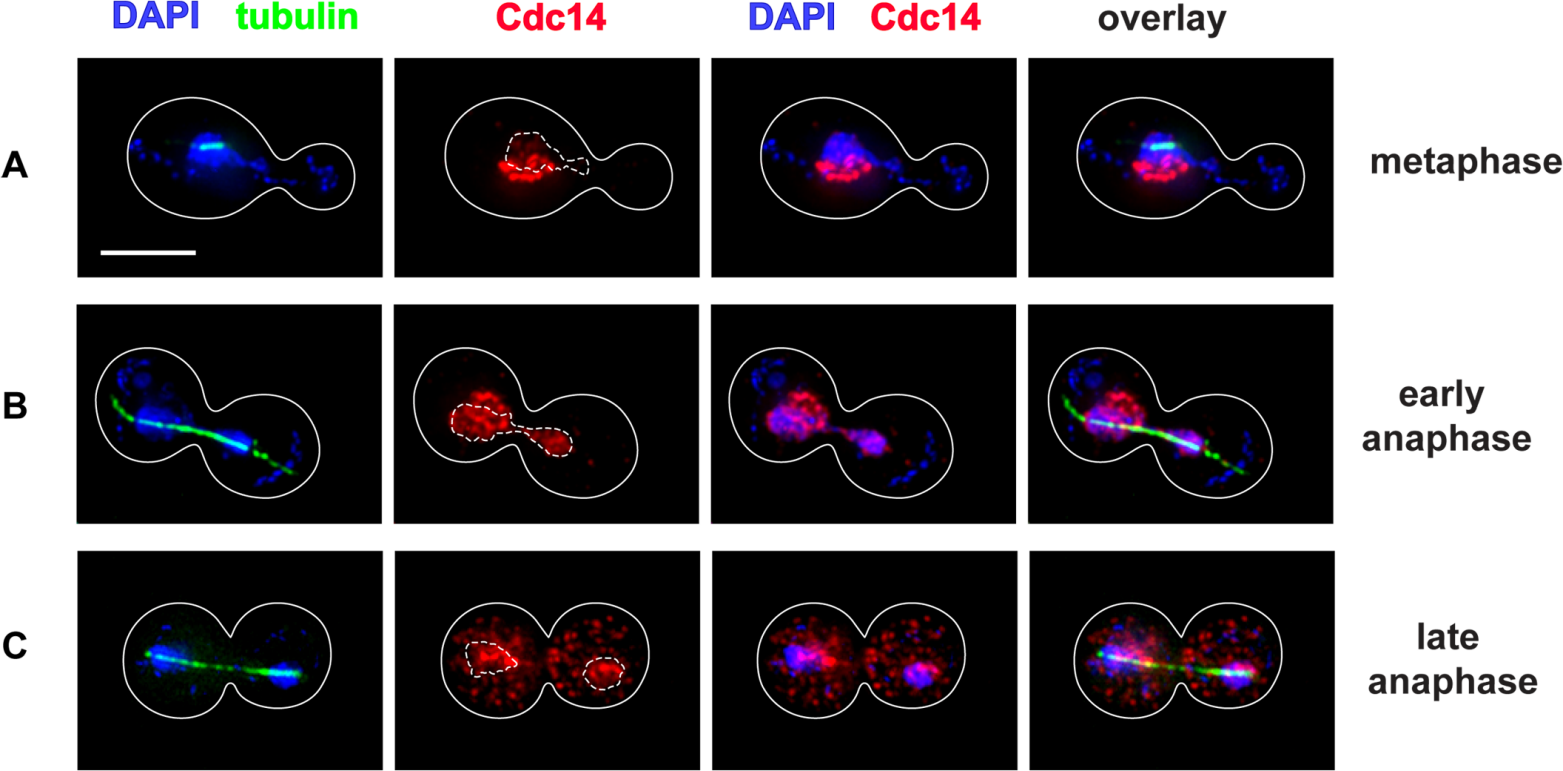
Nuclear-limited Cdc14 release during mitosis. Wild-type cells in log-phase mitosis were stained with DAPI to reveal DNA (in blue) and by indirect immunofluorescence to localize α-tubulin (green) and Cdc14-7Myc (red). Cell cycle stages were identified as described in Materials and Methods. Cell outlines are shown in solid white, and dashed white lines outline the region of strong DAPI-staining (i.e., the nucleus minus the nucleolus, see Materials and Methods). The white bar represents 5 μm. (A) A metaphase cell with Cdc14 sequestered in the nucleolus. (B) An early anaphase cell with Cdc14 released to the nucleus. (C) A late anaphase cell with Cdc14 exported to the cytoplasm.

FEAR has been challenging to study because it is transient, reversible and difficult to distinguish temporally from Cdc14 release by the MEN pathway [8]. Since MEN-activated release of Cdc14 can supplant FEAR, FEAR is usually examined in MEN-defective mutants that have been synchronized and released into the cell cycle. To avoid this experimental intervention, we examined Cdc14 localization in asynchronous wild-type cells. Metaphase cells had the expected nucleolar localization of Cdc14 (Fig 1A), while early anaphase cells had indeed released Cdc14 in a nuclear-limited manner (Fig 1B). By late anaphase, Cdc14 had been efficiently exported to the cytoplasm (Fig 1C), indicating MEN activity. The fraction of cells in the population with nuclear-limited Cdc14 was 11% (average of 4 replicates, with a standard deviation of 0.7), indicating that release of Cdc14 to the nucleus alone is a brief part of normal mitosis. To confirm that we could distinguish nuclear from nucleolar Cdc14, we immunostained Cdc14-7Myc and examined its localization relative to the nucleolar protein Net1-6HA and the mutant net1-6cdk-6HA during early anaphase (S1 Fig). As described previously, [13], the *net1-6cdk-6HA* mutant failed to release Cdc14-7Myc from the nucleolus during early anaphase.

**Fig 2:**
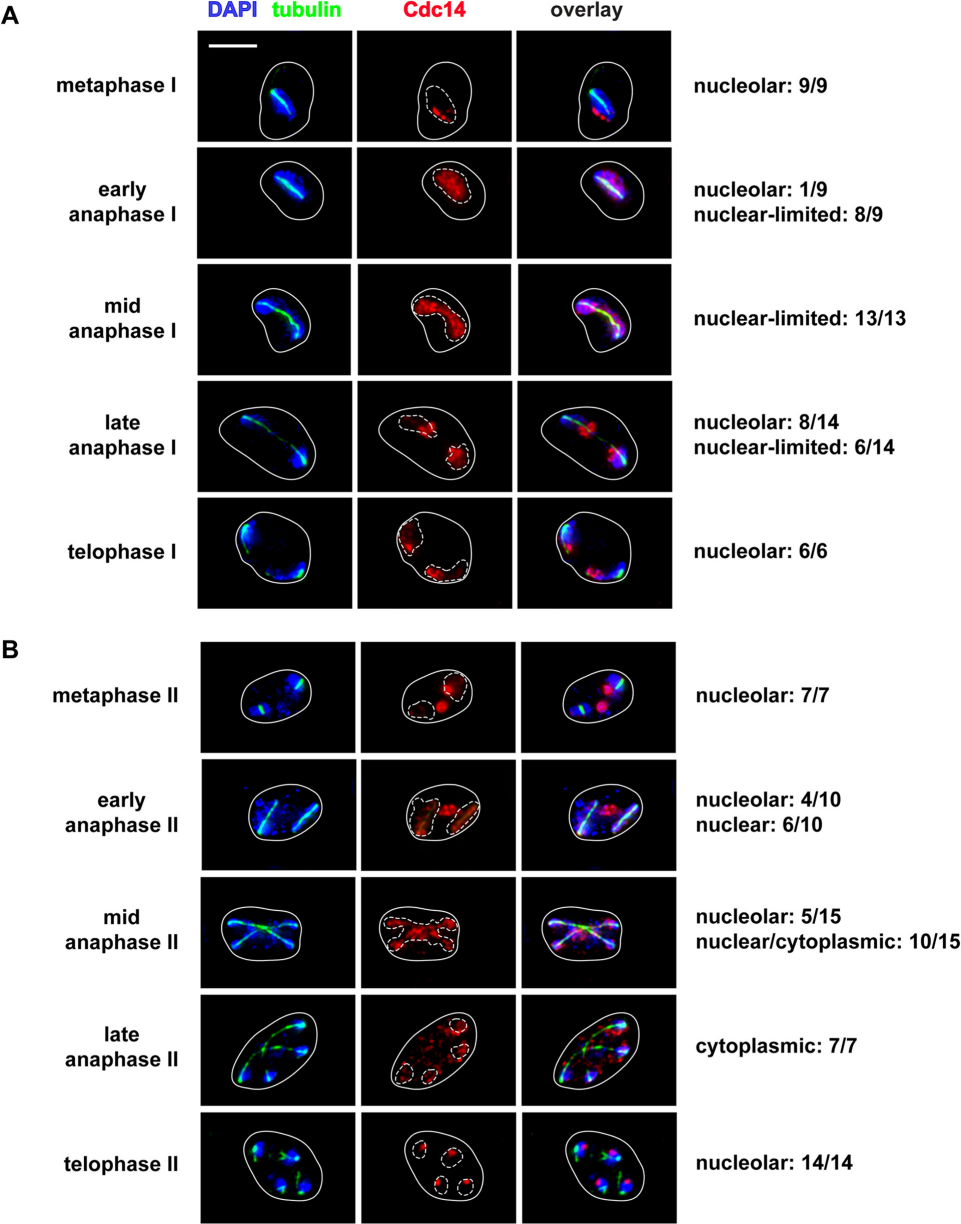
Cdc14-7Myc localization during meiosis I and II. Cdc14-7Myc cells undergoing meiotic nuclear divisions. Images show the most representative Cdc14 localization at each stage of meiosis I and meiosis II. Fractions represent the number of cells with that localization divided by the total number of cells imaged at that stage. Images show Cdc14-7Myc (red), tubulin (green) and DNA (blue). The white bar represents 5 μm.

During meiosis, Cdc14 undergoes two cycles of release from and return to the nucleolus, corresponding to meiosis I and II [39]. Previous studies have shown that FEAR, but not MEN, genes are required for exit from meiosis I [40]. We found that Cdc14 release in meiosis I was limited to the nucleus and therefore very similar to the pattern we saw in early anaphase of mitosis (Fig 2A). Cdc14 persisted in the nucleus until late anaphase I of meiosis, and then began to return to the nucleolus (Fig 2A). In meiosis II, Cdc14 was progressively released into the nucleus and cytoplasm and only fully released to the cytoplasm by late anaphase II (Fig 2B), in a manner similar to MEN-mediated release and nuclear export of Cdc14 in mitosis. Therefore FEAR, as defined cytologically, appears to be limited to the nucleus in both mitosis and meiosis I.

### Stage-specific mitotic arrest points distinguish FEAR from MEN

To distinguish mitotic FEAR from MEN, we arrested cells at specific points from metaphase through mitotic exit and characterized Cdc14 localization in these populations. In response to spindle inhibition by nocodazole, cells activate a series of mechanisms to delay cell cycle progression [41]. The metaphase spindle assembly checkpoint, acting through Mad2, inhibits the ubiquitin ligase APC^Cdc20^, preventing degradation of mitotic cyclins and Pds1 securin as well as inhibiting Esp1 separase to block sister-chromatid separation and arrest cells at metaphase [42]. PP2A^Cdc55^ has two inhibitory roles during arrest. First, like Mad2, it is required to prevent Pds1 degradation; second, it antagonizes Net1 phosphorylation, through which it is thought to prevent FEAR [14, 15]. The Bub2-dependent spindle orientation checkpoint inhibits Cdc14 release by MEN and prevents metaphase, anaphase and mitotic exit [15, 22]. Therefore, while wild-type cells arrest at metaphase in response to nocodazole, *mad2Δ* mutant cells pass through metaphase, *cdc55Δ* cells pass through metaphase and FEAR, and *bub2Δ* cells pass through metaphase, anaphase and mitotic exit.

**Fig 3:**
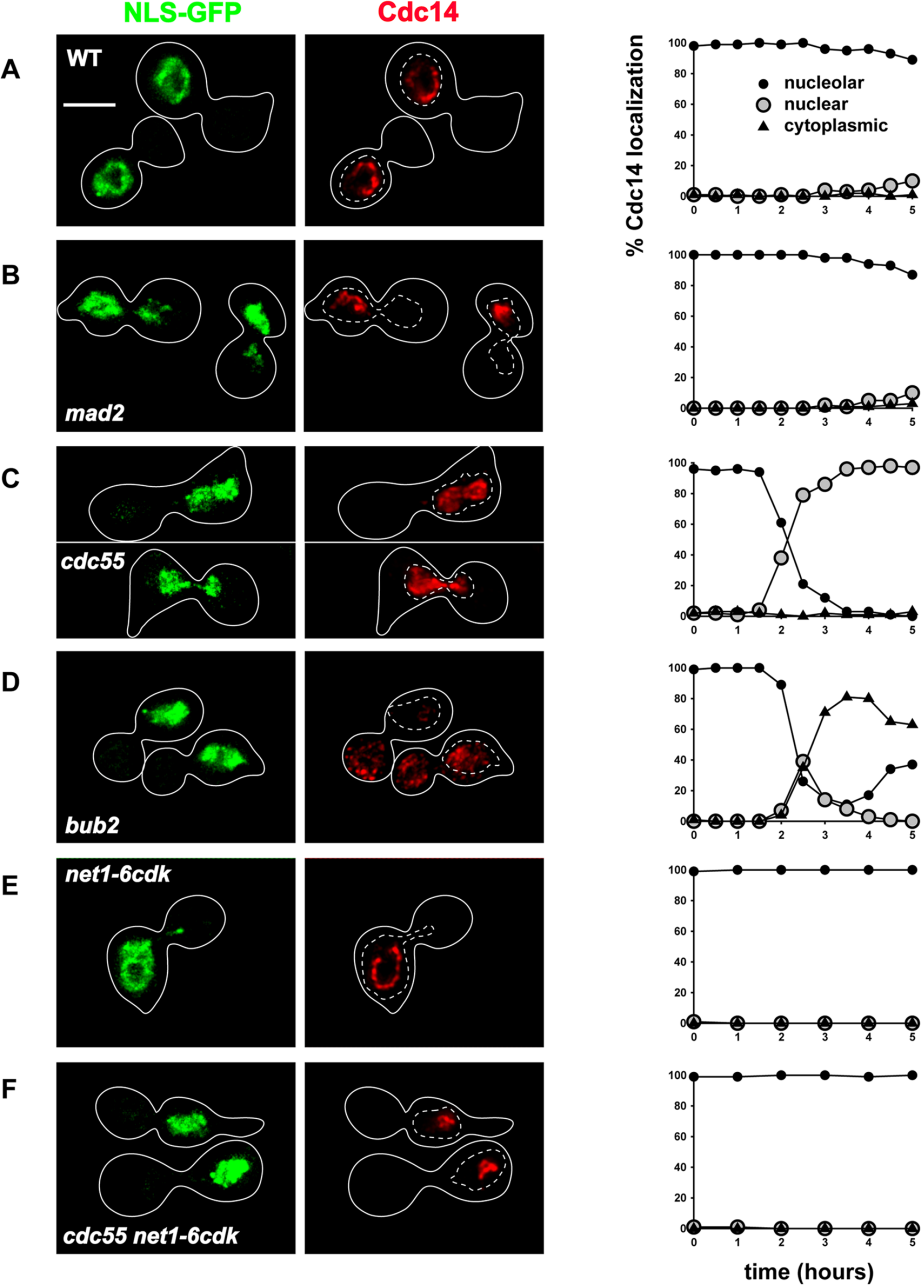
Mitotic arrest reveals nuclear-limited Cdc14 release by the FEAR pathway. See Materials and Methods for the mitotic growth, synchronization and nocodazole-induced arrest procedure. Images of arrested cells were collected 3.5 hours after release from G_1_ arrest into nocodazole. Nuclear-localized GFP is shown in green and Cdc14-7Myc protein in red; the white bar represents 5 μm. Cell outlines are shown in solid white, and nuclei in dashed white lines. Cdc14 was scored as nucleolar (filled circles), nuclear (open circles) or cytoplasmic (triangles). (A) Wild-type cells with Cdc14 sequestered in the nucleolus. (B) *mad2Δ* cells with Cdc14 in the nucleolus. (C) *cdc55Δ* cells with Cdc14 released into the nucleus only. (D) *bub2Δ* cells with Cdc14 exported to the cytoplasm. (E) *net1-6cdk* cells with Cdc14 in the nucleolus. (F) *cdc55Δ net1-6cdk* cells with Cdc14 in the nucleolus.

To accumulate cells at each of the mitotic arrest points, wild type, *mad2Δ*, *cdc55Δ* and *bub2Δ* cells were synchronized in G_1_ phase with α-factor and released into medium containing nocodazole (S2 Fig). Wild-type cells maintained Cdc14 in the nucleolus throughout the time course, as expected for full mitotic arrest (Fig 3A). *mad2Δ* cells, as they reached metaphase, appeared to briefly disperse Cdc14 to the nucleus (Fig 3B). The dispersal of Cdc14 may reflect the abrupt loss of sister chromatid cohesion known to occur in *mad2Δ* cells, and consequent decondensation of the nucleolus. However, Cdc14 was rapidly restored to a tight nucleolar pattern and the *mad2Δ* cells reached mitotic arrest with strictly nucleolar Cdc14 (Fig 3B). In contrast, *cdc55* cells fully and stably released Cdc14 to the nucleus, with Cdc14 undetectable in the cytoplasm (Fig 3C). As expected, *bub2Δ* cells released Cdc14 from the nucleolus and then exported it to the cytoplasm (Fig 3D), indicating mitotic exit. Later in the time course, *bub2Δ* cells began to relocalize Cdc14 to the nucleolus, consistent with previous work showing that they proceed to G_1_ phase during nocodazole-induced arrest [15].

If *cdc55Δ* cells release Cdc14 strictly through the FEAR pathway during mitotic arrest, then the release should be suppressed by the *net1-6cdk* mutation, which prevents FEAR but not MEN [13]. We tested this prediction and Cdc14 release was indeed completely inhibited in nocodazole-arrested *net1-6cdk* control and *cdc55Δ net1-6cdk* cells throughout at least our 5-hour time course (Fig 3F and 3E). If the *net1-6cdk* mutation had not been fully inhibitory to FEAR, then released Cdc14 might have accumulated in *cdc55Δ net1-6cdk* cells during prolonged arrest. However, there was no detectable release of Cdc14, consistent with the *net1-6cdk* mutation being severely inhibitory to FEAR.

One known phenotype of both *cdc55Δ* and *mad2Δ* mutants during mitotic arrest is the release and activation of Esp1, leading to degradation of cohesin [15, 43] and presumably also making Esp1 competent to initiate FEAR. The observation that *cdc55Δ* cells undergo sustained FEAR but *mad2Δ* cells do not, suggests that the release of physiological levels of Esp1 alone is insufficient for a complete and stable release of Cdc14, at least in the context of nocodazole-induced mitotic arrest.

### Mitotic APC^Cdh1^ substrate levels are stable during FEAR

One critical role of released Cdc14 is dephosphorylation of the APC ubiquitin-ligase substrate-specificity factor Cdh1, which activates the E3 ubiquitin ligase APC^Cdh1^ to direct the degradation of numerous mitotic substrates [12, 44–46]. It has not yet been determined whether Cdc14 released by the FEAR pathway can activate APC^Cdh1^. To investigate this possibility, we assayed the stability of several well-studied APC^Cdh1^ substrate proteins in nocodazole-arrested *cdc55Δ* cells by the same arrest-release procedure used for the protein stability assays presented in Fig 3. These substrates include the APC substrate specificity factor Cdc20, the major mitotic cyclin Clb2, the polo-like kinase Cdc5, and the aurora kinase homolog Ipl1 [12, 46–49].

**Fig 4:**
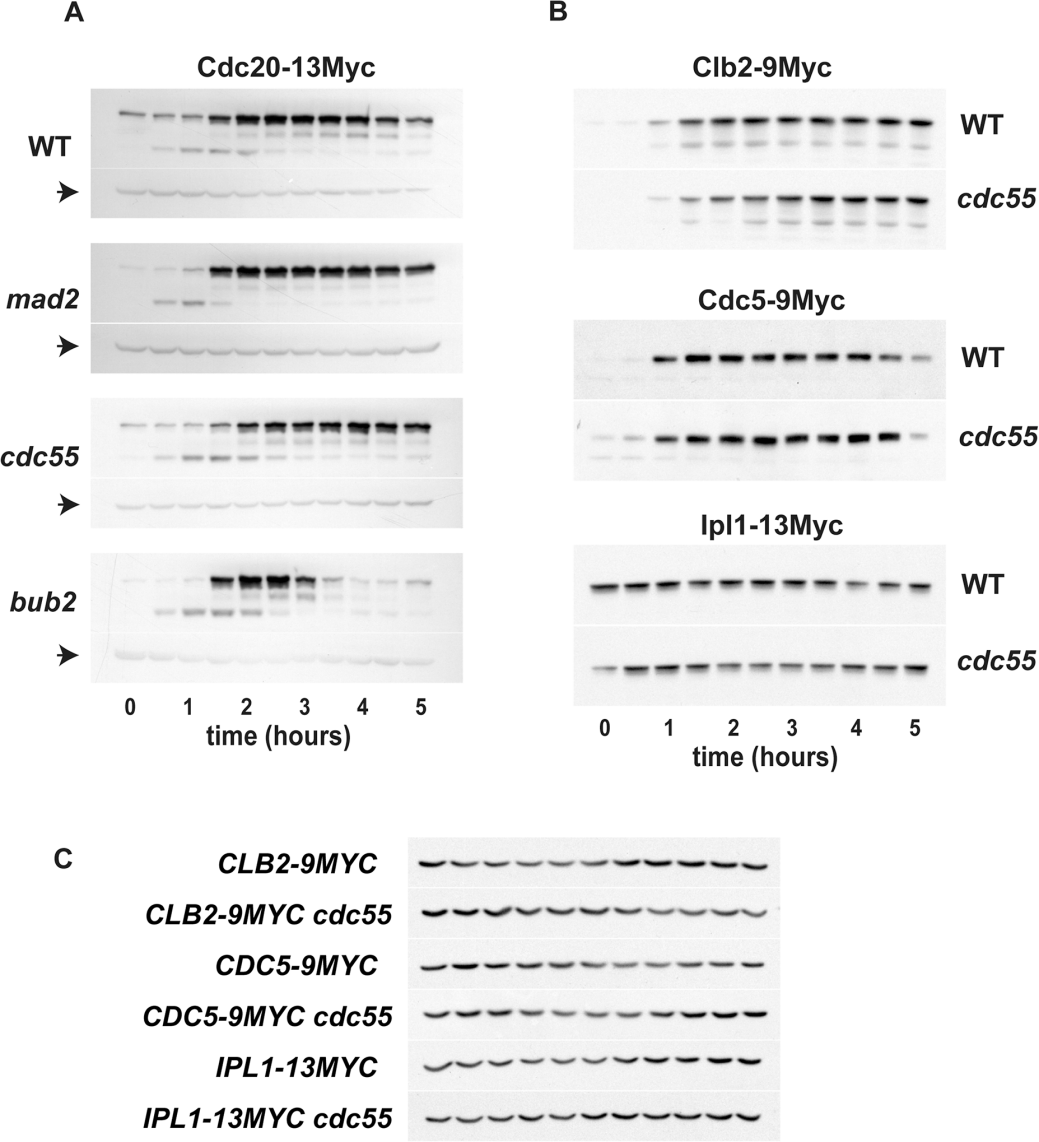
Substrates of APC^Cdh1^ are stable during FEAR. Cells were synchronized and arrested in mitosis as described for Fig 3 (see S2 Fig for cell synchrony data). (A) Cdc20-13Myc protein stability in wild-type, *mad2Δ*, *cdc55Δ* and *bub2Δ* cells. β-tubulin loading controls are indicated with arrows. (B) Stability of Clb2-9Myc, Cdc5-9Myc and Ipl1-13Myc proteins in wild-type and *cdc55Δ* cells. (C) β-actin loading controls for Fig 3B.

Passage through metaphase alone, exemplified by *mad2Δ* cells, did not decrease the level of Cdc20 (Fig 4A). However, *bub2Δ* cells arrested in nocodazole were able to substantially degrade Cdc20 upon activation of MEN (Fig 4A), indicating that the *bub2Δ* cells had initiated mitotic exit, and that nocodazole itself does not interfere with activation of APC^Cdh1^. Levels of Cdc20, Clb2, Cdc5 and Ipl1 were steady in *cdc55Δ* cells during arrest (Fig 4A and 4B). The only exception to the stability of these proteins was the partial degradation of Cdc5 in both *cdc55Δ* and wild-type cells at the last time point, probably due to some cells escaping the arrest. Note that, unlike the other APC^Cdh1^ substrates, Ipl1 protein was present during α-factor arrest, consistent with a previous report that it is not fully degraded during the cell cycle [49]. We have previously reported partial degradation of Clb2 by nocodazole-arrested *cdc55Δ* cells [15], but in light of these additional experiments, it is likely that degradation was due to a slight decay of the arrest state. In summary, even robust release of Cdc14 to the nucleus by the FEAR pathway is insufficient to activate APC^Cdh1^, probably because Cdh1 is sequestered in the cytoplasm during most of mitosis and only imported into the nucleus during mitotic exit [25]. It seems unlikely that FEAR contributes to mitotic exit by activating Sic1 transcription, since Swi5, the key transcription factor for Sic1, is cytoplasmic until mitotic exit and is thought to require dephosphorylation by Cdc14 in order to enter the nucleus [27, 50, 51]. However, we did not directly investigate this possibility.

In summary, restriction of Cdc14 to the nucleus during anaphase may be a mechanism for sharpening the distinction between anaphase and mitotic exit. We suggest that the confinement of Cdc14 to the nucleus precludes its direct participation in mitotic exit functions, such as Clb2 destruction, Sic1 transcription, and cytokinesis, because these events require Cdc14 to be in the cytoplasm [3, 11, 52].

### Deletion of Cdc55 permits FEAR in the absence of Slk19 and Spo12

**Fig 5:**
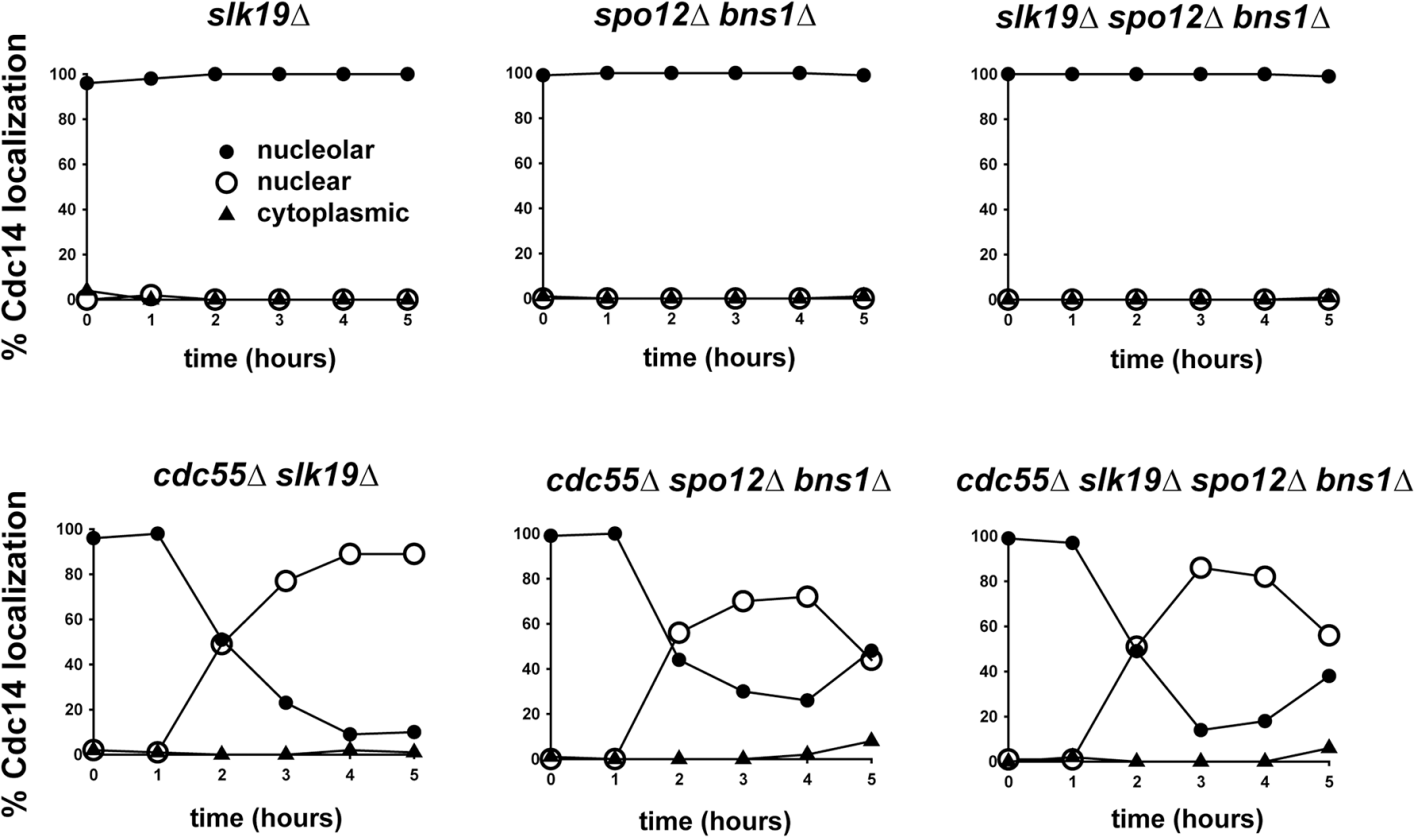
Deletion of Cdc55 bypasses FEAR loss-of-function mutants. Cells were synchronized and arrested in mitosis as described for Figs 3 and 4 (see S2 Fig for synchrony data). Cdc14 protein was scored as nucleolar (black circles), nuclear (gray circles) or cytoplasmic (triangles).

It is thought that Slk19, together with Esp1, primarily stimulates FEAR by downregulating PP2A^Cdc55^, though it might also do so by activating mitotic Cdk to phosphorylate Spo12 [7, 14]. If Slk19 is required to activate Cdk, then FEAR should be impaired in a *slk19Δ cdc55Δ* double mutant. Instead, Cdc14 was fully and stably released into the nucleus during nocodazole-induced mitotic arrest, indicating that Slk19 is dispensable for activation of FEAR in the absence of PP2A^Cdc55^ (Fig 5).

The requirement for Spo12 in activation of FEAR during mitotic arrest was similarly tested, but a *spo12Δ bns1Δ cdc55Δ* triple mutant was used to eliminate the possibility that the *SPO12* homolog *BNS1* (Bypasses the Need for Spo12) [53] would activate FEAR. During mitotic arrest, Cdc14 was released into the nucleus in *cdc55Δ spo12Δ bns1Δ* cells, though less efficiently than in *cdc55Δ slk19Δ*, indicating that deletion of Cdc55 largely bypasses the need for Spo12 and Bns1 in FEAR (Fig 5). However, Cdc14 partially returned to the nucleolus during mitotic arrest, suggesting that Spo12 (and/or Bns1) is required to maintain Cdc14 release. Cdc14 release in *cdc55Δ slk19Δ spo12Δ bns1Δ* and *cdc55Δ SLK19 spo12Δ bns1Δ* cells was similar (Fig 5). Therefore, the *spo12Δ bns1Δ* double mutation is epistatic to deletion of Slk19 for the maintenance of Cdc14 release.

In summary, our findings indicate that deletion of Cdc55 permits the release of Cdc14 from the nucleolus in the absence of Slk19 and Spo12, at least during nocodazole-induced mitotic arrest. However, it appears that Spo12 and/or Bns1 are required to maintain Cdc14 in the released nuclear state, at least during this artificially-induced mitotic arrest.

### The *net1-cdk* mutation does not compromise, but rather enhances MEN activity

Cdc14 released by the FEAR pathway is thought to “prime” MEN activity, thereby increasing the efficiency of mitotic exit [8, 9], and to perform important anaphase functions such as the stabilization of microtubules and spindle midzone structure [8, 19, 20, 54, 55].

The putative contribution of FEAR to mitotic exit has been supported by extensive negative genetic interactions between FEAR and MEN pathway mutations [9, 13, 15, 56]. In particular, there are many previous reports of negative genetic interactions between the *slk19Δ* or *spo12Δ* mutations and MEN mutations [9, 57, 58]. These interactions are believed to reflect the independent contributions of FEAR and MEN to mitotic exit, such that reduction of FEAR under conditions of low MEN activity causes a failure to exit mitosis.

We felt the *net1-6cdk* mutation was a good reagent to test the functional role of FEAR in MEN activation for several reasons. First, our cytological studies show that *net1-6cdk* reduces FEAR to at least the limit of detection of our indirect immunofluorescence microscopy (Figs 1 and 2). Second, the mutation is more specific than the previously-studied FEAR mutations. Finally, even if *net1-6cdk* is a hypomorphic FEAR mutation, the partial loss of FEAR activity should be detectable by its negative effect on the MEN. A corollary prediction is that deletion of *CDC55* should suppress MEN mutations, since cells lacking Cdc55 are known to be hyperactive for Cdc14 release [15, 59, 60]. Indeed, it has been reported that deletion of *CDC55* suppresses the lethality of a *spo12Δ lte1Δ* double mutant, possibly by increasing the early anaphase release of Cdc14 [15].

For these experiments, we analyzed the genetic interactions of *net1-6cdk* and *cdc55Δ* mutations with conditionally lethal MEN mutations representing most of the pathway, including Cdc14 itself. The *lte1Δ* MEN mutation is conditionally lethal at low temperature and also known to be synthetic lethal at 30°C with several FEAR mutants, including *slk19Δ* and *spo12Δ* [9]. The *tem1-3*, *cdc15-2*, *dbf2-2*, *mob1-77* and *cdc14-1* MEN mutations are conditionally lethal at elevated temperatures.

To establish the general validity of our approach, we first confirmed the previously-reported synthetic lethality of *slk19Δ* and *spo12Δ* with *lte1Δ* by dissection of *slk19Δ/+ lte1Δ/+* and *spo12Δ/+ lte1Δ/+* heterozygous diploids. No *slk19Δ lte1Δ* or *spo12Δ lte1Δ* double mutants were recovered from the dissection of 25 tetrads of each diploid strain, while we expected ~25 double mutants to be recovered if they were viable. Likewise, we were unable to recover *slk19Δ tem1-3* or *spo12Δ tem1-3* double mutants from the dissection of 27 and 26 tetrads, respectively, when germinated at 23°C. Double mutants of *slk19Δ* or *spo12Δ* with *cdc15-2* or *dbf2-2* were also recovered at much lower than expected frequencies (data not shown), but we did obtain double-mutants for further study.

**Fig 6:**
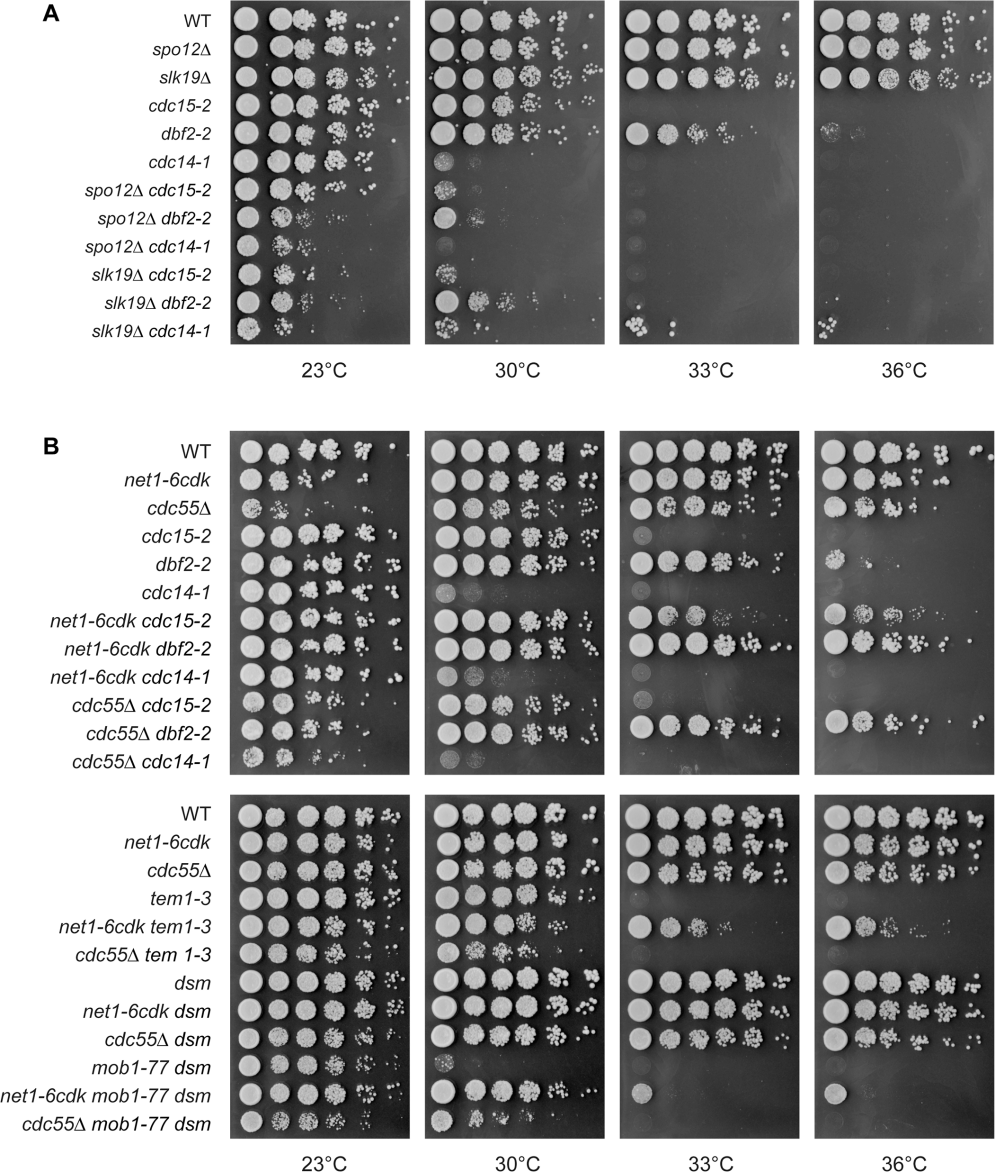
Genetic interactions between FEAR and MEN mutations. Interactions of FEAR mutations with *lte1Δ* were determined by the viability of double-mutant haploid spores germinated at 30°C. To assay interactions with high-temperature-sensitive MEN mutations, cells with the indicated genotypes were grown to saturation in YPD liquid medium at 23°C and a 10-fold dilution series was plated on YPD agar. The plates were incubated at the indicated temperatures for 24–48 before being photographed. (A) Interactions of *slk19Δ* and *spo12Δ* with MEN mutations. (B) Interactions of *net1-6cdk* and *cdc55Δ* with MEN mutations. The W303 strain background carries a dominant suppressor of *mob1-77* temperature-sensitivity at an unknown locus, which we refer to as *DSM*. We backcrossed the recessive (non-suppressing) allele *dsm* along with *mob1-77* into our W303 strains a minimum of five times to eliminate the suppressor.

**Fig 7:**
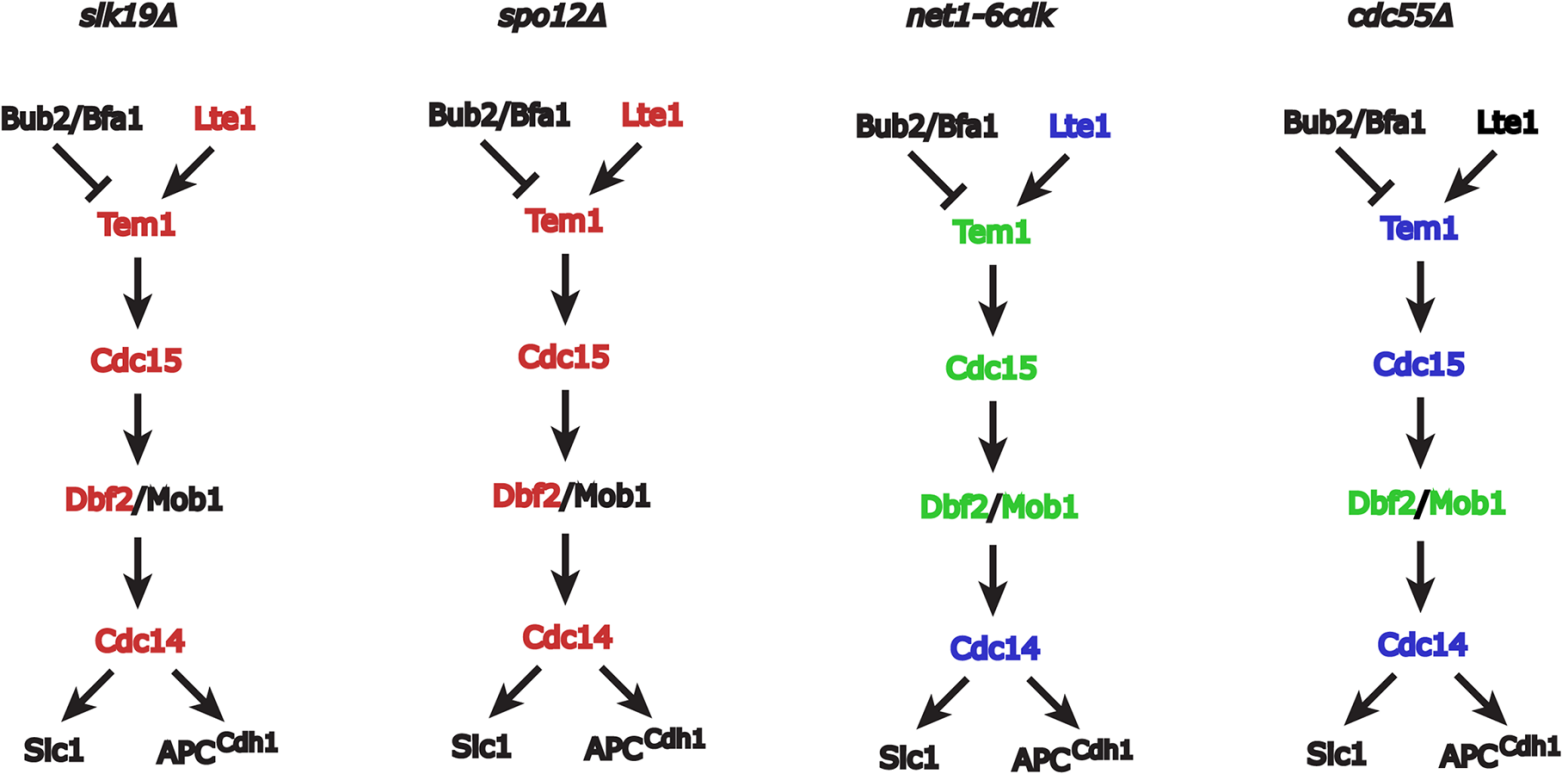
Summary of interactions between FEAR and MEN mutations. MEN proteins in red had mutations that interacted negatively with the indicated FEAR mutation, enhancing temperature-sensitivity. MEN proteins in green had mutations that interacted positively with the FEAR mutation, suppressing temperature-sensitivity. MEN proteins in blue had mutations that did not significantly interact with the indicated FEAR mutation. The interactions of proteins in black were not determined in this study.

In a serial dilution assay, the *slk19Δ* and *spo12Δ* mutations interacted negatively with *cdc15-2*, *dbf2-2* and *cdc14-1*, resulting in lower permissive temperatures for the double mutants than for the single MEN mutants (Fig 6A). A partial exception to this pattern was the emergence of suppressor colonies in *slk19Δ cdc14-1* double mutants on a background of inviable cells. Slk19 is required for accurate chromosome segregation, so these suppressors may arise due to aneuploidy [61], but we did not investigate that possibility. In summary, the *slk19Δ* and *spo12Δ* mutations interacted negatively with all members of the MEN pathway that we tested (Fig 7).

To assay the interaction between *net1-6cdk* and *lte1Δ* mutations, we dissected 25 tetrads from a strain heterozygous for *net1-6cdk* and *lte1Δ*. All *net1-6cdk lte1Δ* double mutants were viable (24 recovered/24 expected), and the mitotic doubling time of a *net1-6cdk lte1Δ* double mutant was indistinguishable from the *lte1Δ* single mutant (Table 1). Therefore, unlike other FEAR mutations, *net1-6cdk* failed to interact negatively with *lte1Δ*. We further analyzed the interactions of *net1-6cdk* with the MEN mutations *tem1-3*, *cdc15-2*, *dbf2-2*, *mob1-77* and *cdc14-1*. To our surprise, *net1-6cdk* suppressed the temperature-sensitivity of all the mutations except *cdc14-1*, with which we detected no interaction (Figs 6B and 7). The *net1-6cdk* mutation enhances MEN activity but does not bypass the essentiality of Cdc14, so it may indeed act by influencing Cdc14 activity.

**Table 1:** Mitotic doubling times.

genotype	temp. (°C)	time (minutes)^a^	% of wild-type
wild-type	30	84.8 +/- 0.7	100
*slk19Δ*	30	98.2 +/- 2.1	116
*spo12Δ*	30	88.6 +/- 1.0	105
*net1-6cdk*	30	89.9 +/- 1.4	106
*lte1Δ*	30	93.7 +/- 1.9	111
*net1-6cdk lte1Δ*	30	94.8 +/- 1.3	112
wild-type	25	113.6 +/- 2.3	100
*net1-6cdk*	25	121.0 +/- 1.5	107

^a^ Values represent the means +/- SD.

Our findings disagree with an earlier report that the *net1-6cdk* mutation exacerbates the temperature-sensitivity of *cdc15-2* and *dbf2-2* mutants [13]. However, the *net1-6cdk* allele used in that study had a C-terminal fusion to a TEV protease site followed by nine repeats of the Myc epitope. We found that the *net1-6cdk-TEV-9Myc* allele interacted negatively with several MEN mutations (our unpublished results), so we performed all of our genetic experiments with a *net1-6cdk* allele that had only the phosphorylation site changes.

To determine whether the *cdc55Δ* mutation could contribute to MEN activity, we analyzed the genetic interactions of *cdc55Δ* with the MEN mutations *tem1-3*, *cdc15-2*, *dbf2-2*, *mob1-77* and *cdc14-1*. The interactions of *cdc55Δ* with *tem1-3*, *cdc15-2* and *cdc14-1* were relatively neutral, but *cdc55Δ* partially suppressed the temperature-sensitivity of *dbf2-2* and *mob1-77* (Figs 6B and 7). Dbf2 and Mob1 form a kinase complex, and Dbf2 has recently been shown to directly oppose the phosphatase PP2A at the entry into mitosis [62]. Our observation of the suppression of *dbf2-2* and *mob1-77* by deletion of the PP2A subunit Cdc55 may reflect the opposing activities of the Dbf2/Mob1 kinase and PP2A^Cdc55^ phosphatase during mitotic entry rather than mitotic exit. In any case, the genetic interactions between *cdc55Δ* and the MEN temperature-sensitive alleles present a mixed pattern rather than overall suppression. Therefore, hyperactivation of FEAR alone is not sufficient to compensate for reduced MEN activity.

### FEAR is not required for efficient progression through mitotic exit

In support of the hypothesis that FEAR is required for efficient mitotic exit, it is often mentioned that *slk19Δ* and *spo12Δ* mutants, in addition to delaying the early anaphase release of Cdc14 by 10–15 minutes, take longer than normal to complete a mitotic cell cycle [8]. We measured the effects of the *slk19Δ*, *spo12Δ* and *net1-6cdk* mutations on overall cell cycle duration. Relative to wild-type cells, *slk19Δ*, *spo12Δ* and *net1-6cdk* cells growing at 30°C were delayed by 13, 4 and 5 minutes, respectively (Table 1). We believe the additional delay observed in *slk19Δ* cells is primarily due to a role for Slk19 in anaphase spindle elongation, distinct from FEAR [63, 64].

FEAR is thought to be required for the timely completion of spindle growth and disassembly, resolution of nucleolar cohesion, activation of mitotic exit, and cytokinesis [9, 19, 65, 66]. If the delay of these events is specifically the consequence of losing FEAR, then not only the anaphase release of Cdc14, but also spindle formation and disassembly, nuclear division, export of Cdc14 from the nucleus and completion of cell division should be delayed in *net1-6cdk* cells comparably to what has been reported for *slk19Δ* and *spo12Δ* mutants. Conversely, if the delays in anaphase and mitotic exit previously reported for FEAR mutants are not evident in *net1-6cdk* cells, then we might conclude that FEAR *per se* is not required for timely passage through these cell cycle transitions.

**Fig 8:**
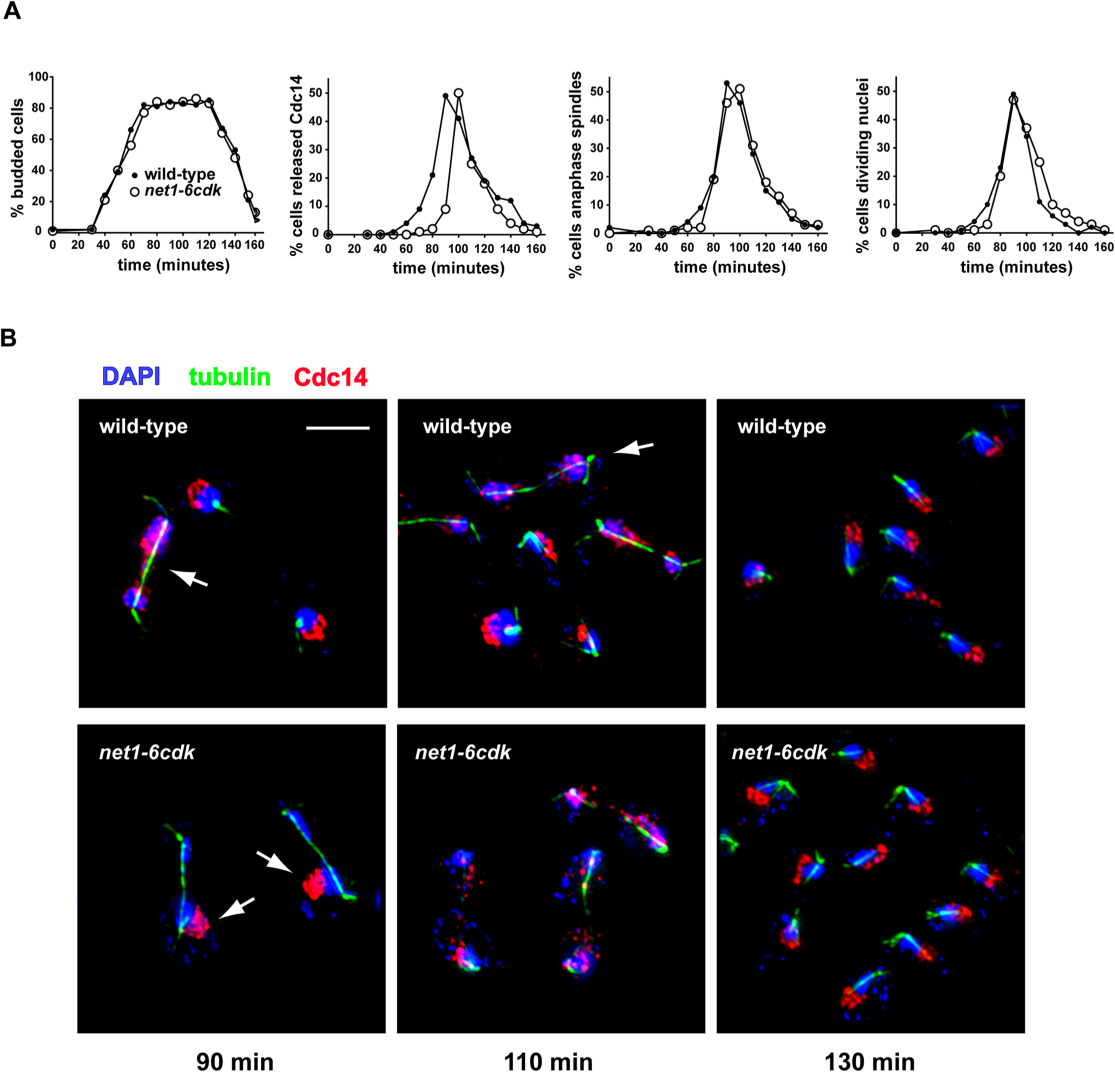
*net1-6cdk* cells progress efficiently through anaphase and mitotic exit. Synchronized cells growing at 25°C. (A) Quantitative analysis of landmarks of mitotic progression. B) Indirect immunofluorescence of Cdc14-3Myc protein in red, spindle staining in green and DAPI-stained DNA in blue. At 90 minutes, an early anaphase wild-type cell undergoing FEAR (arrow) and two *net1-6cdk* cells that have not released Cdc14 (arrows); at 110 minutes, a wild-type cell undergoing MEN release of Cdc14 (arrow) and three net1-6cdk cells undergoing MEN release; at 130 minutes both wild-type and *net1-6cdk* cells with Cdc14 returned to the nucleolus.

Using synchronized wild-type and *net1-6cdk* cells released from G_1_ phase arrest, we determined the kinetics of a set of events characteristic of anaphase and mitotic exit. The events we looked at were Cdc14 release and return to the nucleolus, anaphase spindle formation and breakdown, nuclear division, bud formation and cell separation (i.e. cell division). The *net1-6cdk* cells did indeed delay Cdc14 release by approximately 10–12 minutes (Fig 8A), similar to what has been reported in studies of other FEAR mutants [7, 9, 13]. Unlike wild-type cells, however, *net1-6cdk* mutant cells consistently began anaphase spindle elongation before releasing Cdc14 (Fig 8B, 90 min.), evincing the classic FEAR phenotype. As the mitotic spindle elongated into the bud, *net1-6cdk* cells efficiently released Cdc14 and promptly exported it from the nucleus (Fig 8A and 8B, 110 min.), indicating timely activation of the MEN. The nuclear export of Cdc14 was followed by its rapid return to the nucleolus (Fig 8A and 8B, 130 min.). In fact, Cdc14 was re-sequestered in the nucleolus at least as quickly in *net1-6cdk* cells as in the wild-type controls. Bud formation, indicating entry into the cell cycle, and bud separation, indicating the completion of cytokinesis, occurred with similar timing in wild-type and *net1-6cdk* cells (Fig 8A). The *net1-6cdk* cells also underwent anaphase spindle formation/disassembly and nuclear division, two physically linked processes, at the same time as wild-type cells. Among the events we measured, only the completion of nuclear division was slightly delayed in *net1-6cdk* mutant cells. We believe this brief delay is most likely the result of inefficient rDNA separation, a known phenotype of FEAR mutants [8, 66, 67]. Our findings (and unpublished observations of rDNA segregation) are consistent with previous work showing that the timing of anaphase spindle formation in *net1-6cdk* and *spo12Δ* cells is normal, while both mutations delay rDNA segregation to the same degree [13]. In summary, FEAR may be required for efficient rDNA segregation, but it is not required for timely mitotic exit and progression to G_1_ phase.

## Discussion

The FEAR pathway has been correlated with a wide variety of anaphase events, most importantly anaphase spindle stabilization, rDNA segregation and timely mitotic exit [8]. The model has been based entirely on studies in the yeast *Saccharomyces cerevisiae* using mutations in genes, including *ESP1*, *CDC5*, *SLK19* and *SPO12*, with known or potential non-FEAR roles [8, 63]. The advantage in using the *net1-6cdk* mutation is that it inhibits Cdc14 release without compromising the potential non-FEAR activities of upstream pathway members.

The PP2A mutant *cdc55Δ* is a complementary tool to *net1-6cdk*. Deletion of *CDC55* allows prolonged activation of the pathway, albeit under mitotic arrest conditions that may not fully represent the behavior of cycling cells. We have used the *net1-6cdk* and *cdc55Δ* mutations to alternatively inhibit or activate FEAR. As evidence that this is a sound strategy, we find that the *cdc55Δ* mutation allows strong FEAR-activated Cdc14 release even in the absence of key FEAR pathway members (Fig 5), and yet that release is fully inhibited by the *net1-6cdk* mutation (Fig 3).

These genetic reagents have allowed us to address two outstanding issues regarding FEAR. First, we asked whether FEAR, defined cytologically as the Cdc14 release itself, is truly limited to the nucleus, and whether that limitation has functional implications. Second, we asked whether FEAR is truly required for the timely execution of anaphase events and potentiation of MEN activity.

### Nucleolar limitation of FEAR

We find that FEAR is limited to the nucleus in both mitosis and meiosis I. Even prolonged activation of FEAR does not promote the nuclear export of Cdc14 (Fig 3) or the degradation of APC^Cdh1^ substrates as required for mitotic exit (Fig 4). Since APC^Cdh1^ substrates are stable during prolonged FEAR activation, FEAR-released Cdc14 probably does not directly antagonize Cdk activity during normal growth, at least for the purpose of mitotic exit. Restriction to the nucleus is therefore an important constraint on FEAR activity.

### Functions of FEAR

What about the functional contributions of FEAR to anaphase and mitotic exit? The current model holds that when FEAR is compromised, anaphase events should be delayed and MEN activity reduced. Below we discuss the implications of our findings for understanding the functions of early anaphase Cdc14 release.

#### Anaphase spindle formation

The hypothesis that FEAR modulates anaphase spindle elongation has been difficult to reconcile with the modest effects of FEAR deletion mutants, and the literature contains some contradictory evidence. Several reports suggest that Cdc14 is a crucial for early anaphase spindle elongation, and that Cdc14 acts in this role by dephosphorylating targets at the spindle midzone [19, 20]. However, in a control experiment in which all detectable Cdc14 was depleted, only the *initiation* of spindle growth was modestly delayed, while the kinetics of anaphase spindle formation were relatively normal (Pereira and Schiebel, 2003, Fig 1). In addition, the canonical FEAR mutant *spo12Δ* forms anaphase spindles with wild-type kinetics [13].

Based on our limited analysis, anaphase spindles form and break down with normal timing in the *net1-6cdk* mutant (Fig 8). In mitotic *net1-6cdk* cells, we also consistently saw spindle elongation before Cdc14 had been released from the nucleolus (Figs 1 and 6). While the spindles we observe may not be fully normal, the release of Cdc14 is evidently not required for the scheduling of anaphase spindle formation, elongation or breakdown.

The connection of FEAR to spindle regulation has largely been based on experiments in which overexpression of an Esp1 catalytic mutant during metaphase arrest triggered ectopic Cdc14 release [16, 18–20]. However, a more recent study shows that this overexpression primarily stimulates Cdc14 release through cohesin degradation, and requires mitotic spindles to do so [21]. The spindle-dependent nature of this release suggests that Esp1 overexpression during mitotic arrest activates not FEAR, but the MEN, and that it does so by allowing spindle pole translocation through the bud neck, an event which is thought to lead to MEN activation [22]. Our findings are consistent with this interpretation. In the *net1-6cdk* mutant, anaphase spindles form, grow and translocate through the bud neck with almost wild-type kinetics, and only then are followed by MEN-activated Cdc14 release (Fig 8). In retrospect, the published images of Cdc14 release activated by Esp1 overexpression, which can be seen to cause the export of Cdc14 from the nucleus to the cytoplasm (Sullivan and Uhlmann, 2003, Fig 1), very likely represent MEN activation.

#### rDNA segregation

We did not re-investigate this role of FEAR, since the *net1-6cdk* mutation has already been shown to delay rDNA segregation [13], but our observations (unpublished data) were consistent with an rDNA segregation delay similar to other FEAR mutants. Therefore, delayed rDNA segregation emerges as a unifying phenotype of the known FEAR mutants, suggesting it may indicate a core function of the proteins that have been implicated in FEAR. Broadly speaking, there are two ways FEAR might promote rDNA segregation. One is that the release of Cdc14, as believed, activates events that lead to rDNA cohesion being lost. The other is that nucleolar Cdc14 inhibits rDNA segregation, and it is the removal of Cdc14 from the nucleolus that is required. Our experiments do not allow us to distinguish between these two alternatives, but it should in theory be possible to do so by expressing, in addition to wild-type Cdc14, a form of Cdc14 that would be constitutively retained within the nucleolus. Finally, we should consider that the rDNA segregation delay seen in FEAR mutants may be an indirect consequence of interfering with rDNA composition or with the RENT complex, and not indicative of a specific functional role.

#### MEN potentiation

Current models of anaphase regulation have been founded on the assumption that that FEAR is an activating event in mitotic exit [9]. Our genetic analysis suggests that, at least with respect to the MEN, the opposite may be true (Figs 6 and 7). Besides suggesting a novel functional interpretation (discussed below), these results support the assumption, implicit in our experiments, that *net1-6cdk* is a severe loss-of-function FEAR mutant. FEAR mutations have been thought to decrease MEN activity. According to the current model, regardless of whether *net1-6cdk* is severely inhibitory or only hypomorphic for FEAR, it should at least partially reduce MEN activity. The fact that *net1-6cdk* actually *increases* MEN pathway activity indicates it is not simply a hypomorphic FEAR mutant.

Because many FEAR mutations are non-lethal, FEAR is implicitly non-essential, but it had been thought that the mitotic exit delay seen in FEAR mutants indicated inefficient MEN activation. Our finding that the *net1-6cdk* mutation actually increases MEN activity (Figs 6 and 7) therefore directly challenges the current understanding of mitotic exit. The simplest interpretation of our result is that maintenance of Cdc14 in the nucleolus during early anaphase actually enhances MEN activity. An important caveat to this interpretation is that the *net1-6cdk* mutation, like *slk19Δ*, *spo12Δ*, and other “FEAR” mutations, may have undescribed pleiotropic effects on mitotic exit independent of the inhibition of FEAR. In light of that caveat, we suggest two alternative interpretations. First, that instead of the release of Cdc14 being required for anaphase progression, perhaps dispersal of Cdc14 by FEAR subtly delays MEN activation until rDNA segregation is under way. This delay could provide time for the completion of anaphase and ensure that nucleolar division and mitotic exit occur in the appropriate sequence. Second, FEAR might have no influence on the MEN. Suppression of MEN mutations by the *net1-6cdk* mutation could result from phenotypes unrelated to FEAR. In either case, we think we have presented strong evidence that FEAR does not potentiate the activity of the MEN.

### Thoughts about future work

The timely passage of *net1-6cdk* cells through anaphase and mitotic exit landmark events (Fig 8) highlights not only the dispensability of FEAR, but the efficiency with which the MEN alone can drive cells through mitotic exit. Therefore, the *net1-6cdk* mutant should be a useful genetic background in which to search for determinants of MEN-specific Cdc14 release. A MEN-specific release mutant should be synthetic lethal with *net1-6cdk*. Identifying these release factors is an important goal for the field, and we hope our insights will help.

Since FEAR *per se* is not required for efficient mitotic exit, we believe mutations such as *slk19Δ* and *spo12Δ*, which delay mitotic exit, have non-FEAR activities that affect mitotic exit. Indeed, since Slk19 is required for anaphase spindle growth independently of its role in FEAR [63], it is likely to affect mitotic exit by directly delaying MEN activation. Likewise, Spo12 is likely to have a FEAR-independent effect on mitotic exit, a possibility hinted at by a long-standing observation concerning *SPO12* gene dosage. Overexpression of Spo12 suppresses many lethal MEN pathway mutations, but not *cdc14-1* [29, 30, 45, 68, 69]. We found that even the full release of Cdc14 during metaphase arrest does not lead to indications of mitotic exit (Figs 3 and 4), and that FEAR does not activate the MEN. Therefore, in order to supplant MEN activity, *SPO12* overexpression probably activates Cdc14 in some way other than simply promoting its release. Our work suggests that new hypotheses for the functions of the FEAR proteins are needed.

The regulation of Cdc14 localization and the molecular circuitry controlling mitotic exit have recently been integrated into quantitative and systems level models [70–73]. To be properly founded and developed, this type of model should be subjected to *in vivo* functional testing when possible. We hope our work will contribute to these emerging models by establishing the physical and functional limitations of FEAR.

## Supporting Information

S1 Fig FEAR localization relative to the nucleolar protein Net1. Cells in log-phase mitosis were stained with DAPI to reveal DNA (in blue) and by indirect immunofluorescence to localize Cdc14-7Myc (red) relative to Net1-6HA or net1-6cdk-6HA (green). The early anaphase cell cycle phase was identified as described in Materials and Methods. Cell outlines are shown in solid white, and dashed white lines outline the region of strong DAPI-staining (i.e., the nucleus minus the nucleolus, see Materials and Methods).
(TIF)

S2 Fig Bud morphology of mitotic time courses. Bud morphology data for synchronous mitotic time courses. Cells were scored as unbudded or budded using a phase contrast microscope. The data are identified by the corresponding figure number in the main text and by genotype.
(TIF)

S1 Table Yeast strains used in this study. (DOC)

S2 Table Yeast strains used by figure and table. (DOCX)
