# Supplementary figures and images for: Cdc14 Early Anaphase Release, FEAR, Is Limited to the Nucleus and Dispensable for Efficient Mitotic Exit
Source: PLoS One. 2015 Jun 19;10(6):e0128604. doi: 10.1371/journal.pone.0128604 (PMC4474866; doi:10.1371/journal.pone.0128604)

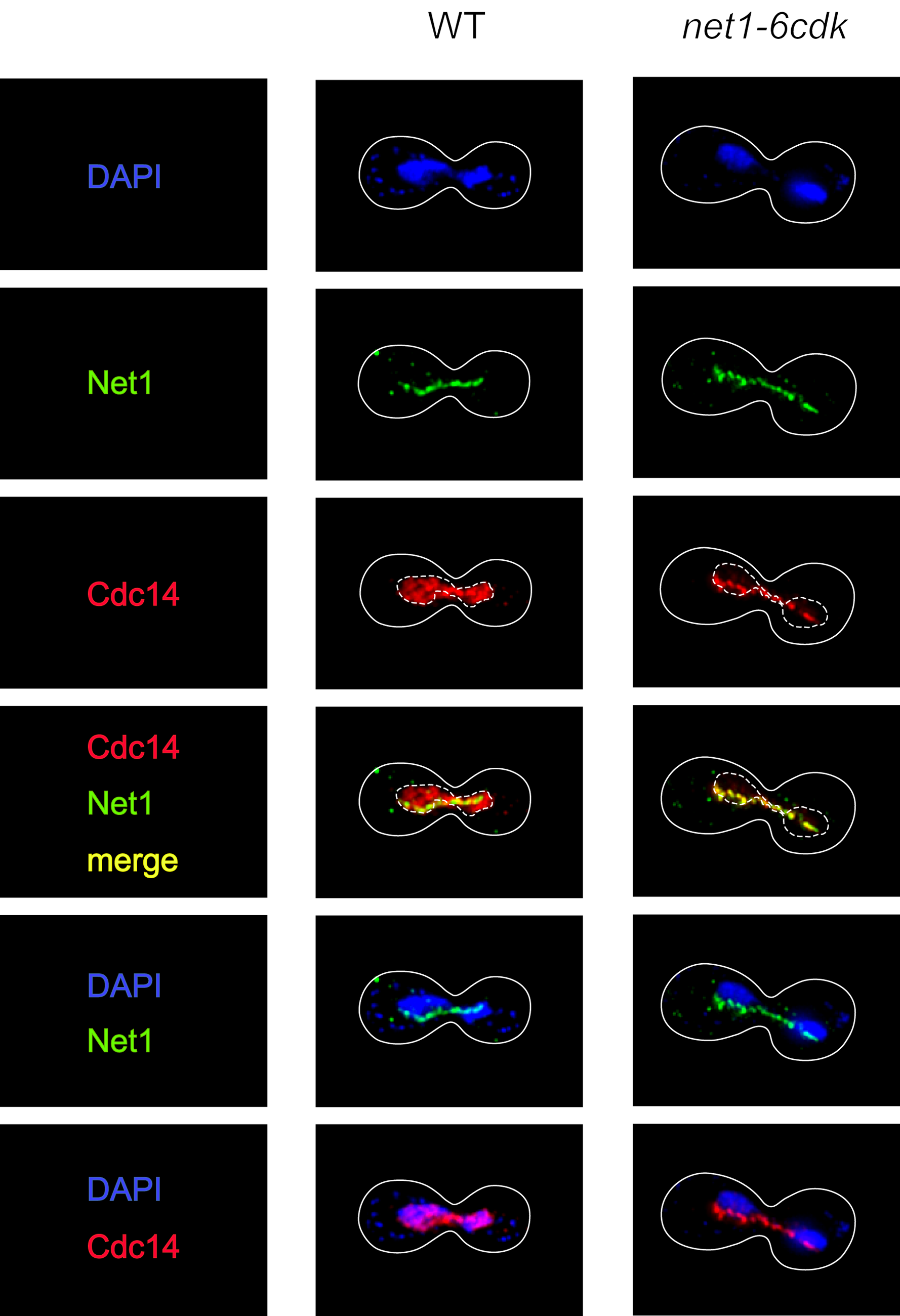

Supplement: S1 Fig — Cells in log-phase mitosis were stained with DAPI to reveal DNA (in blue) and by indirect immunofluorescence to localize Cdc14-7Myc (red) relative to Net1-6HA or net1-6cdk-6HA (green). The early anaphase cell cycle phase was identified as described in Materials and Methods. Cell outlines are shown in solid white, and dashed white lines outline the region of strong DAPI-staining (i.e., the nucleus minus the nucleolus, see Materials and Methods). (TIF) [file pone.0128604.s001.tif]

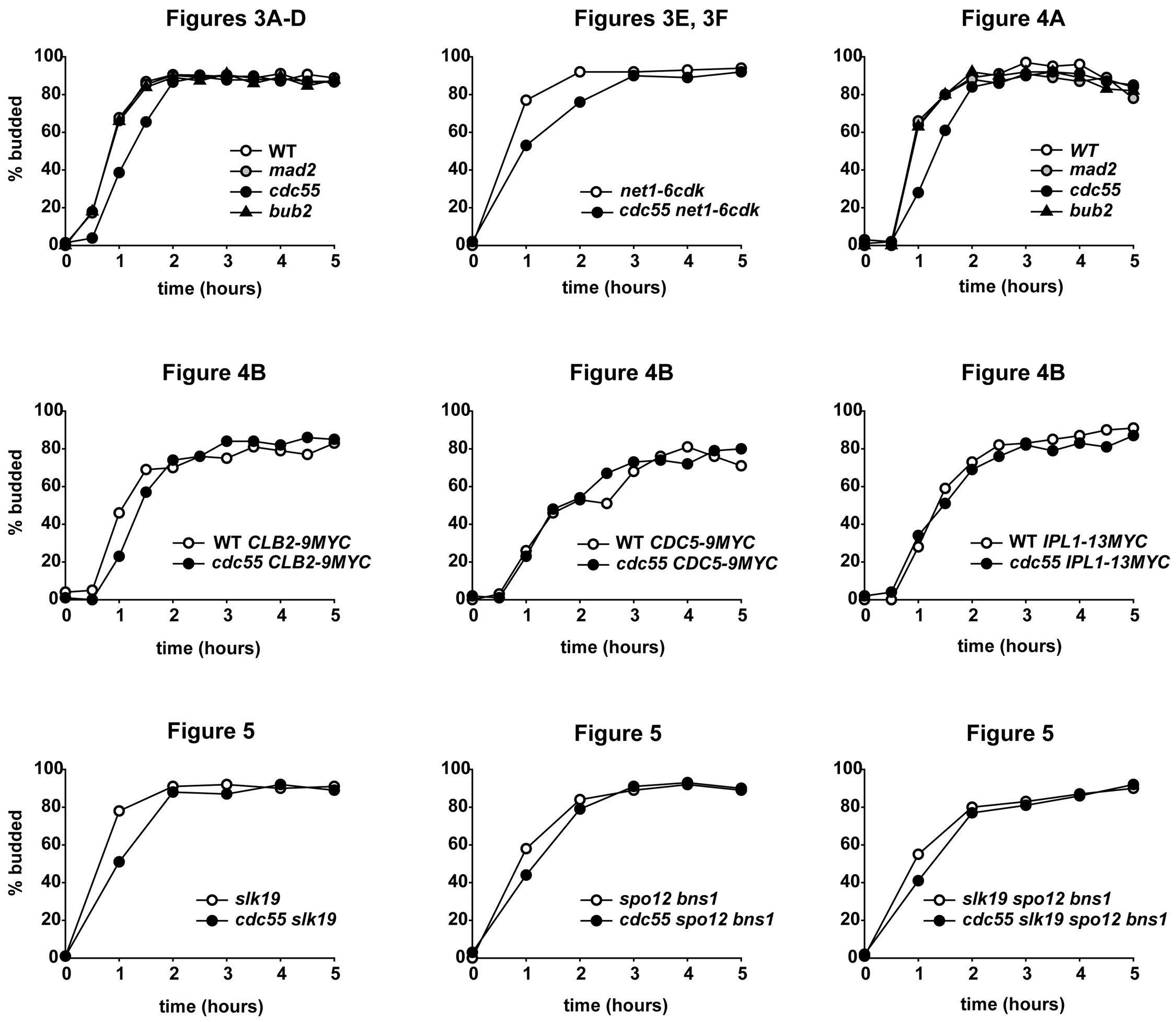

Supplement: S2 Fig — Bud morphology data for synchronous mitotic time courses. Cells were scored as unbudded or budded using a phase contrast microscope. The data are identified by the corresponding figure number in the main text and by genotype. (TIF) [file pone.0128604.s002.tif]
