# Supplementary material for: Cdc14 Early Anaphase Release, FEAR, Is Limited to the Nucleus and Dispensable for Efficient Mitotic Exit
Source: PLoS One. 2015 Jun 19;10(6):e0128604. doi: 10.1371/journal.pone.0128604 (PMC4474866; doi:10.1371/journal.pone.0128604)
Supplement: S1 Table — (DOC) [file pone.0128604.s003.doc]

**S1 Table.** Yeast strain genotypes.

| strain ID | genotype | source |
| --- | --- | --- |
| CMY 675-3C | MAT**a** can1-100 his3-11,15 leu2-3,112 trp1-1 ura3-1 bar1::hisG | this study |
| CMY 813-2C | same as CMY 1448-7D but cdc55::kanMX4 | this study |
| CMY 915-1B | same as CMY 1448-7D but dbf2-2 | this study |
| CMY 920-2D | same as CMY 1448-7D but cdc55::natMX4 | this study |
| CMY 1061-1C | same as CMY 1448-7D but cdc55::natMX4 dbf2-2 | this study |
| CMY 1068-11A | same as CMY 1448-7D but cdc15-2 | this study |
| CMY 1068-24D | same as CMY 1448-7D but cdc55::natMX4 cdc15-2 | this study |
| CMY 1080-9D | same as CMY 1448-7D but cdc55::natMX4 cdc14-1 | this study |
| CMY 1083-4C | same as CMY 1448-7D but cdc55::kanMX4 tem1-3 | this study |
| CMY 1320-4D | same as CMY 1448-7D but cdc55::natMX4 dsm1 | this study |
| CMY 1320-10D | same as CMY 1448-7D but cdc55::natMX4 kanMX4-mob1-77 dsm1 | this study |
| CMY 1320-13A | same as CMY 1448-7D but dsm1 | this study |
| CMY 1448-7D | *MAT***a** *can1-100 his3-11,15 leu2-3,112 trp1-1 ura3-1* | this study |
| CMY 1543-2B | same as CMY 675-3C but *CLB2-9MYC-kanMX6* | this study |
| CMY 1611-6D | same as CMY 675-3C but *CDC14-7MYC-HIS3MX6* | this study |
| CMY 1611-13D | same as CMY 675-3C but *cdc55::natMX4 CDC14-7MYC-HIS3MX6* | this study |
| CMY 1612-4A | same as CMY 675-3C but *bub2::kanMX4 CDC14-7MYC-HIS3MX6* | this study |
| CMY 1613-4B | same as CMY 675-3C but *mad2::kanMX4 CDC14-7MYC-HIS3MX6* | this study |
| CMY 1631-6D | same as CMY 675-3C but *cdc55::natMX4 CDC5-9MYC-kanMX6* | this study |
| CMY 1631-9D | same as CMY 675-3C but *CDC5-9MYC-kanMX6* | this study |
| CMY 1633-3D | same as CMY 675-3C but *cdc55::natMX4 CLB2-9MYC-kanMX6* | this study |
| CMY 1634-1C | same as CMY 675-3C but *cdc55::natMX4 IPL1-13MYC-HIS3MX6* | this study |
| CMY 1634-16B | same as CMY 675-3C but *IPL1-13MYC-HIS3MX6* | this study |
| CMY 1673-1A | same as CMY 675-3C but *net1-6cdk-kanMX4 CDC14-7MYC-HIS3MX6* *net1-6cdkS-kanMX4* | this study |
| CMY 1673-14D | same as CMY 675-3C but *cdc55::natMX4 net1-6cdk-kanMX4*  *CDC14-7MYC-HIS3MX6* | this study |
| CMY 1724-16A | same as CMY 675-3C but *cdc55::natMX4 slk19::bleMX4*  *CDC14-7MYC-HIS3MX6* | this study |
| CMY 1724-37A | same as CMY 675-3C but *slk19::bleMX4 CDC14-7MYC-HIS3MX6* | this study |
| CMY 1781-3A | same as CMY 675-3C but *spo12::hphMX4 bns1::kanMX4*  *CDC14-7MYC-HIS3MX6* | this study |
| CMY 1781-12D | same as CMY 675-3C but *slk19::bleMX4 spo12::hphMX4 bns1::kanMX4 CDC14-7MYC-HIS3MX6* | this study |
| CMY 1781-29B | same as CMY 675-3C but *cdc55::natMX4 slk19::bleMX4 spo12::hphMX4 bns1::kanMX4 CDC14-7MYC-HIS3MX6* | this study |
| CMY 1781-53C | same as CMY 675-3C but *cdc55::natMX4 spo12::hphMX4 bns1::kanMX4 CDC14-7MYC-HIS3MX6* | this study |
| CMY 1882-4C | same as CMY 675-3C but CDC14-7MYC-HIS3MX6 NET1-6HA-kanMX6 | this study |
| CMY 1883-4C | same as CMY 675-3C but CDC14-7MYC-HIS3MX6 net1-6cdk-6HA-kanMX6 | this study |
| CMY 2051-13D | same as CMY 1448-7D but kanMX4-mob1-77 dsm1 | this study |
| CMY 2150 | *MAT***a***/MATα can1-100/+ his3-11,15/his3-11,15 leu2-3,112/+ trp1-1/+ ura3-1/+ CDC14-7MYC-HIS3MX6/CDC14-7MYC-HIS3MX6* | this study |
| CMY 2210 | *MAT***a***/MATα ade2-1/+ can1-100/can1-100 his3-11,15/his3-11,15*  *leu2-3,112/leu2-3,112 trp1-1/trp1-1 ura3-1/ura3-1* | this study |
| CMY 2309-4D | same as 1448-7D but *spo12::natMX4* | this study |
| CMY 2324-16C | same as 1448-7D but *lte1::kanMX4* | this study |
| CMY 2331-1C | same as 1448-7D but *slk19::hphMX4* | this study |
| CMY 2379-13C | same as 675-3C but *CDC14-7MYC-HIS3MX6*  *leu2:LEU2-NLS-tetR-GFP* | this study |
| CMY 2377-45D | same as 675-3C but *mad2::kanMX4 CDC14-7MYC-HIS3MX6 leu2:LEU2-NLS-tetR-GFP* | this study |
| CMY 2378-19D | same as 675-3C but *cdc55::natMX4 CDC14-7MYC-HIS3MX6*  *leu2:LEU2-NLS-tetR-GFP* | this study |
| CMY 2379-61C | same as 675-3C but *bub2::kanMX4 CDC14-MYC-HIS3MX6*  *leu2:LEU2-NLS-tetR-GFP* | this study |
| CMY 2378-19C | same as 675-3C but *net1-6cdk-kanMX4 CDC14-7MYC-HIS3MX6 leu2:LEU2-NLS-tetR-GFP* | this study |
| CMY 2378-9D | same as 675-3C but *cdc55::natMX4 net1-6cdk-kanMX4*  *CDC14-7MYC-HIS3MX6 leu2:LEU2-NLS-tetR-GFP* | this study |
| CMY 2470-8D | same as 1448-7D but *net1-6cdk-hphMX4* | this study |
| CMY 2570-3D | same as 1448-7D but *cdc14-1* | this study |
| CMY 2570-20A | same as 1448-7D but *net1-6cdk-hphMX4 cdc14-1* | this study |
| CMY 2728 | same as CMY 2210 but *lte1::kanMX4/+ +/net1-6cdk-hphMX4* | this study |
| CMY 2728-5D | same as 1448-7D but *net1-6cdk-hphMX4 lte1::kanMX4* | this study |
| CMY 2753-2C | same as CMY 675-3C but *ade2-1* *CDC14-3MYC-kanMX6* | this study |
| CMY 2753-4B | same as CMY 675-3C but *ade2-1* *CDC14-3MYC-kanMX6 net1-6cdk-hphMX4* | this study |
| CMY 2895 | same as CMY 2210 but *lte1::kanMX4/+ +/slk19::hphMX4* | this study |
| CMY 2896 | same as CMY 2210 but *lte1::kanMX4/+ +/spo12::natMX4* | this study |
| CMY 3098-11A | same as 1448-7D but *net1-6cdk-hphMX4 dbf2-2* | this study |
| CMY 3100-2A | same as 1448-7D but *net1-6cdk-hphMX4 cdc15-2* | this study |
| CMY 3122-17C | same as 1448-7D but *tem1-3* | this study |
| CMY 3122-13C | same as 1448-7D but *net1-6cdk-hphMX4 tem1-3* | this study |
| CMY 3129-1B | same as CMY 1448-7D but net1-6cdk-hphMX4 kanMX4-mob1-77 dsm1 | this study |
| CMY 3129-13A | same as CMY 1448-7D but net1-6cdk-hphMX4 dsm1 | this study |
| CMY 3152 | same as CMY 2210 but *+/spo12::natMX4 tem1-3/+* | this study |
| CMY 3153 | same as CMY 2210 but *+/spo12::natMX4 cdc15-2/+* | this study |
| CMY 3153-5A | same as CMY 1448-7D but *spo12::natMX4 cdc15-2* | this study |
| CMY 3154 | same as CMY 2210 but *+/spo12::natMX4 dbf2-2/+* | this study |
| CMY 3154-29D | same as CMY 1448-7D but *spo12::natMX4 dbf2-2* | this study |
| CMY 3155 | same as CMY 2210 but *+/spo12::natMX4 cdc14-1/+* | this study |
| CMY 3155-14B | same as CMY 1448-7D but *spo12::natMX4 cdc14-1* | this study |
| CMY 3156 | same as CMY 2210 but *+/slk19::hphMX4 tem1-3/+* | this study |
| CMY 3157 | same as CMY 2210 but *+/slk19::hphMX4 cdc15-2/+* | this study |
| CMY 3157-18B | same as CMY 1448-7D but *slk19::hphMX4 cdc15-2* | this study |
| CMY 3158 | same as CMY 2210 but *+/slk19::hphMX4 dbf2-2/+* | this study |
| CMY 3158-22C | same as CMY 1448-7D but *slk19::hphMX4 dbf2-2* | this study |
| CMY 3159 | same as CMY 2210 but *+/slk19::hphMX4 cdc14-1/+* | this study |
| CMY 3159-2B | same as CMY 1448-7D but *slk19::hphMX4 cdc14-1* | this study |
| CMY 8950-2-4  (A364a) | *MAT***a** *can1 cyh2 his3Δ1 leu2-3,112 trp1-289 ura3-52 bar1::loxP*  *CDC20-12MYC-kanMX6* | Burke lab |
| CMY 8950-3-2  (A364a) | same as CMY 8950-2-4 but *cdc55::loxP-natMX4-loxP* | Burke lab |
| CMY 8951-4-2  (A364a) | same as CMY 8950-2-4 but *bub2::URA3* | Burke lab |
| CMY 8952-9-3 (A364a) | same as CMY 8950-2-4 but *mad2::URA3* | Burke lab |
