# Supplementary material for: Cdc14 Early Anaphase Release, FEAR, Is Limited to the Nucleus and Dispensable for Efficient Mitotic Exit
Source: PLoS One. 2015 Jun 19;10(6):e0128604. doi: 10.1371/journal.pone.0128604 (PMC4474866; doi:10.1371/journal.pone.0128604)
Supplement: S2 Table — (DOCX) [file pone.0128604.s004.docx]

**S2 Table. Yeast strains used by figure and table**

**Figure 1**

CMY1611-6D

**Figure 2**

CMY 2150

**Figure 3**

Images are of the following strains:

wild-type CMY 2379-13C

*mad2Δ* CMY 2377-45D

*cdc55Δ* CMY 2378-19D

*bub2Δ* CMY 2379-61C

*net1-6cdk* CMY 2378-19C

*cdc55Δ net1-6cdk* CMY 2378-9D

Quantification of Cdc14 localization in Figure 3 used the following strains:

wild-type CMY 1611-6D

*mad2Δ* CMY 1613-4B

*cdc55Δ* CMY 1611-13D

*bub2Δ* CMY 1612-4A

*net1-6cdk* CMY 1673-1A

*cdc55Δ net1-6cdk* CMY 1673-14D

**Figure 4A**

wild-type CMY 8950-2-4

*mad2Δ* CMY 8952-9-3

*cdc55Δ* CMY 8950-3-2

*bub2Δ* CMY 8951-4-2

**Figure 4B**

*CLB2-9MYC* CMY 1543-2B

*cdc55Δ CLB2-9MYC* CMY 1633-3D

CDC5-9MYC CMY 1631-9D

*cdc55Δ CDC5-9MYC* CMY 1631-6D

*IPL1-13MYC* CMY 1634-16B

*cdc55Δ IPL1-13MYC*  CMY 1634-1C

**Figure 5**

*slk19Δ* CMY 1724-37A

*cdc55Δ slk19Δ* CMY 1724-16A

*spo12Δ bns1Δ* CMY 1781-3A

*cdc55Δ spo12Δ bns1Δ* CMY 1781-53C

*slk19Δ* *spo12Δ bns1Δ* CMY 1781-12D

*cdc55Δ* *slk19Δ* *spo12Δ bns1Δ* CMY 1781-29B

**Figure 6 and accompanying genetic experiments**

The following diploids were dissected to test genetic interactions with *lte1Δ*:

*slk19Δ/+ lte1Δ /+* CMY 2895

*spo12Δ/+ lte1Δ/+* CMY 2896

*net1-6cdk/+ lte1Δ/+* CMY 2728

The following diploids were dissected to test genetic interactions with *tem1-3*:

*slk19Δ/+ tem1-3/+* CMY 3156

*spo12Δ/+ tem1-3/+* CMY 3152

**Figure 6A**

wild-type CMY 1448-7D

*spo12Δ* CMY 2309-4D

*slk19Δ* CMY 2331-1C

*cdc15-2* CMY 1068-11A

*dbf2-2* CMY 915-1B

*cdc14-1* CMY 2570-3D

*spo12Δ cdc15-2* CMY 3153-5A

*spo12Δ dbf2-2* CMY 3154-29D

*spo12Δ cdc14-1* CMY 3155-14B

*slk19Δ cdc15-2* CMY 3157-18B

*slk19Δ dbf2-2* CMY 3158-22C

*slk19Δ cdc14-1* CMY 3159-2B

**Figure 6B, set 1**

wild-type CMY 1448-7D

*net1-6cdk* CMY 2470-8D

*cdc55Δ* CMY 920-2D

*cdc15-2* CMY 1068-11A

*dbf2-2* CMY 915-1B

*cdc14-1* CMY 2570-3D

*net1-6cdk cdc15-2* CMY 3100-2A

*net1-6cdk dbf2-2* CMY 3098-11A

*net1-6cdk cdc14-1* CMY 2570-20A

*cdc55Δ cdc15-2* CMY 1068-24D

*cdc55Δ dbf2-2* CMY 1061-1C

*cdc55Δ cdc14-1* CMY 1080-9D

**Figure 6B, set 2**

wild-type CMY 1448-7D

*net1-6cdk* CMY 2470-8D

*cdc55Δ* CMY 813-2C

*tem1-3* CMY 3122-17C

*net1-6cdk tem1-3* CMY 3122-13C

*cdc55Δ tem1-3* CMY 1083-4C

*dsm* CMY 1320-13A

*net1-6cdk dsm* CMY 3129-13A

*cdc55Δ dsm* CMY 1320-4D

*mob1-77 dsm* CMY 2051-13D

*net1-6cdk mob1-77 dsm* CMY 3129-1B

*cdc55Δ mob1-77 dsm* CMY 1320-10D

**Figure 8**

wild-type CMY 2753-2C

*net1-6cdk* CMY 2753-4B

**S1 Figure**

*NET1-6HA* CMY 1882-4C

*net1-6cdk-6HA* CMY 1883-4C

**Table 1**

wild-type CMY 1448-7D

*net1-6cdk-hphMX4* CMY 2470-8D

*lte1::kanMX4* CMY 2324-16C

*net1-6cdk-hphMX4 lte1::kanMX4* CMY 2728-5D
